# Effectiveness of BNT162b2 Vaccine Booster against SARS-CoV-2 Infection and Breakthrough Complications, Israel

**DOI:** 10.3201/eid2805.220141

**Published:** 2022-05

**Authors:** Aharona Glatman-Freedman, Michal Bromberg, Yael Hershkovitz, Hanna Sefty, Zalman Kaufman, Rita Dichtiar, Lital Keinan-Boker

**Affiliations:** Tel Aviv University School of Public Health, Tel Aviv, Israel (A. Glatman-Freedman, M. Bromberg);; Israel Center for Disease Control, Ministry of Health, Ramat Gan, Israel (A. Glatman-Freedman, M. Bromberg, Y. Hershkovitz, H. Sefty, Z. Kaufman, R. Dichtiar, L. Keinan-Boker);; Haifa University School of Public Health, Haifa, Israel (L. Keinan-Boker)

**Keywords:** COVID-19, coronavirus disease, SARS-CoV-2, severe acute respiratory syndrome coronavirus 2, viruses, respiratory infections, zoonoses, BNT162b2, vaccines, Pfizer-BioNTech, Israel

## Abstract

We estimated vaccine effectiveness (VE) of the BNT162b2 (Pfizer-BioNTech, https://www.pfizer.com) booster dose against SARS-CoV-2 infection and reduction of complications (hospitalization, severe disease, and death) among breakthrough cases in persons in Israel >16 years of age for <20 weeks. VE estimates reached 96.8% (95% CI 96.0%–97.5%) for persons 16–59 years of age and 93.1% (95% CI 91.8%–94.2%) for persons >60 years of age on week 3. VE estimates remained at these levels for 8 weeks in the 16–59 age group and 11 weeks in those >60. A slow decline followed, becoming more pronounced in the last 2–3 weeks of evaluation. Estimates in the last week of evaluation were 77.6% (95% CI 68.4%–84.2%) and 61.3% (52.5%–68.4%) for persons 16–59 years and >60 years, respectively. The more pronounced VE decline coincided with rapid increase in Omicron variant activity. Rate reduction of breakthrough complications remained moderate to high throughout the evaluation.

The mass severe acute respiratory syndrome coronavirus 2 (SARS-CoV-2) BNT162b2 (Pfizer-BioNTech, https://www.pfizer.com) vaccination campaign in Israel was associated with a decline in the number of SARS-CoV-2 infections, hospitalizations, and deaths, reaching a nadir by mid-May 2021 (*1*). However, beginning the third week of June 2021, a new rise in the number of SARS-CoV-2 cases was observed, including cases among fully vaccinated persons (*1*,*2*). Waning humoral immune response after the second vaccine dose was then found to be associated with increased incidence of SARS-CoV-2–related infections, hospitalizations, and deaths caused primarily by the B.1.617.2 (Delta) variant (*3*). In response to the increasing illness and deaths, the Israel Ministry of Health (MOH) recommended a third (booster) BNT162b2 vaccine dose for persons for whom at least 5 months had passed after receiving the second vaccine dose (*4*). The elderly and other high-risk groups were prioritized at first (*4*), and other age groups were added rapidly thereafter (*5*). We estimated the booster dose vaccine effectiveness (VE) against SARS-CoV-2 infection and the rate reduction of complications in breakthrough coronavirus disease (COVID-19) cases after the BNT162b2 booster dose in persons >16 years of age, by age group, for up to 20 weeks after receipt of the booster dose.

## Methods

### Study Design

We conducted a retrospective longitudinal cohort study using 2 MOH national repositories: the COVID-19 vaccine repository and the SARS-CoV-2 test repository. The national COVID-19 vaccine repository includes vaccine type, vaccine lot number, and date of dose administration for each person vaccinated in Israel. The national SARS-CoV-2 PCR test database includes the results of each test performed, the date of testing, and the date results were obtained for each person. It also includes the date of hospitalization, severity of illness, and date of death of persons with COVID-19, if applicable. Personal identifiers such as unique personal identity number, age, and sex of each person registered in the repositories (because of PCR testing or vaccination) are included in both databases. We retrieved individual deidentified data from both databases and matched persons by using twice-encrypted unique personal identity numbers.

During the first stage of our study, we determined VE for booster dose vaccine recipients against SARS-CoV-2 infection by using unvaccinated persons as controls. During the second stage, we determined the rate reduction for hospitalizations, severe or critical disease, and deaths among persons who tested positive for SARS-CoV-2 after the booster dose (i.e., breakthrough cases).

**Figure 1:**
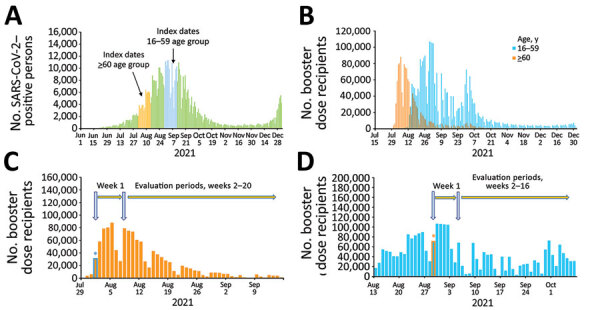
Estimations of effectiveness of BNT162b2 vaccine booster (Pfizer, https://www.pfizer.com) against SARS-CoV-2 infection and breakthrough complications, Israel. A) Epidemic curve of new PCR-confirmed SARS-CoV-2–positive persons, June 1, 2021–January 1, 2022. Index dates are highlighted in orange (for persons >60 years of age) and light blue (for persons 16–59 years of age). B) Daily booster dose recipients by age group. C) Graphic illustration of the booster dose vaccine effectiveness evaluation method for a single cohort of persons >60 years of age that received the booster dose on August 1, 2021. Orange bars represent the number of persons who received the booster dose each day; light blue asterisk represents the date persons >60 years of age included in cohort 1 received the booster dose. D) Graphic illustration of the booster dose vaccine effectiveness evaluation method for a single cohort of persons 16–59 years of age who received the booster dose on August 29, 2021. Light blue bars represent the number of persons who received the booster dose each day; orange asterisk represents the date persons 16–59 years of age included in cohort 1 received the booster dose.

We defined as index dates the dates on which third-dose vaccine recipients in our study received the booster dose (Figure 1, panel A). Booster dose recipients and unvaccinated controls included in each index date represented a single cohort. We performed analyses for persons 16–59 years of age across 14 consecutive cohorts with the index dates August 29, 2021–September 11, 2021. These dates were selected because, by that period, persons 16–59 years of age had already been approved by the MOH to receive the booster dose (Appendix Figure 1). Analyses for persons >60 years of age were performed across 14 consecutive cohorts with index dates occurring during August 1, 2021–August 14, 2021. These dates were chosen for this age group because this group was the first to receive the booster dose (Figure 1, panel B) and because most persons >60 years of age received the third dose before August 29, 2021 (Appendix Figure 1). We followed each cohort through January 1, 2022.

### Estimation of VE

**Figure 2:**
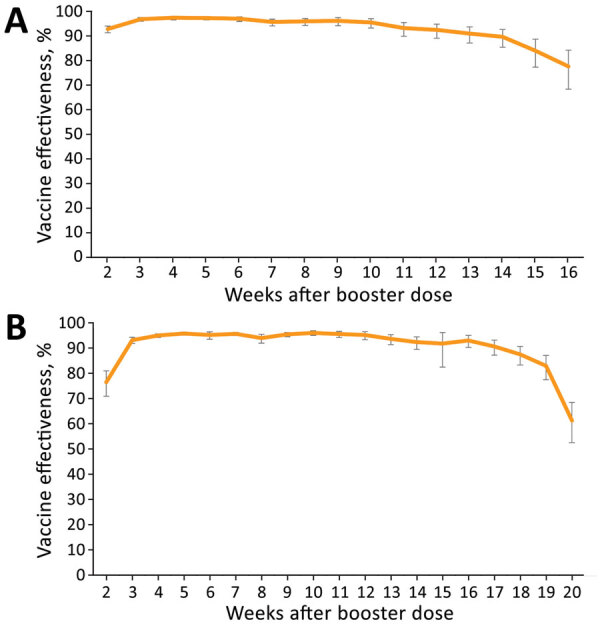
Adjusted vaccine effectiveness against severe acute respiratory syndrome coronavirus 2 infection in persons 16–59 years of age, by week, September 6, 2021–January 1, 2022 (A), and >60 years of age, by week, August 9, 2021–January 1, 2022 (B), Israel. Adjustments were performed for sex, age, and epidemiologic week. Error bars represent 95% CIs.

**Figure 3:**
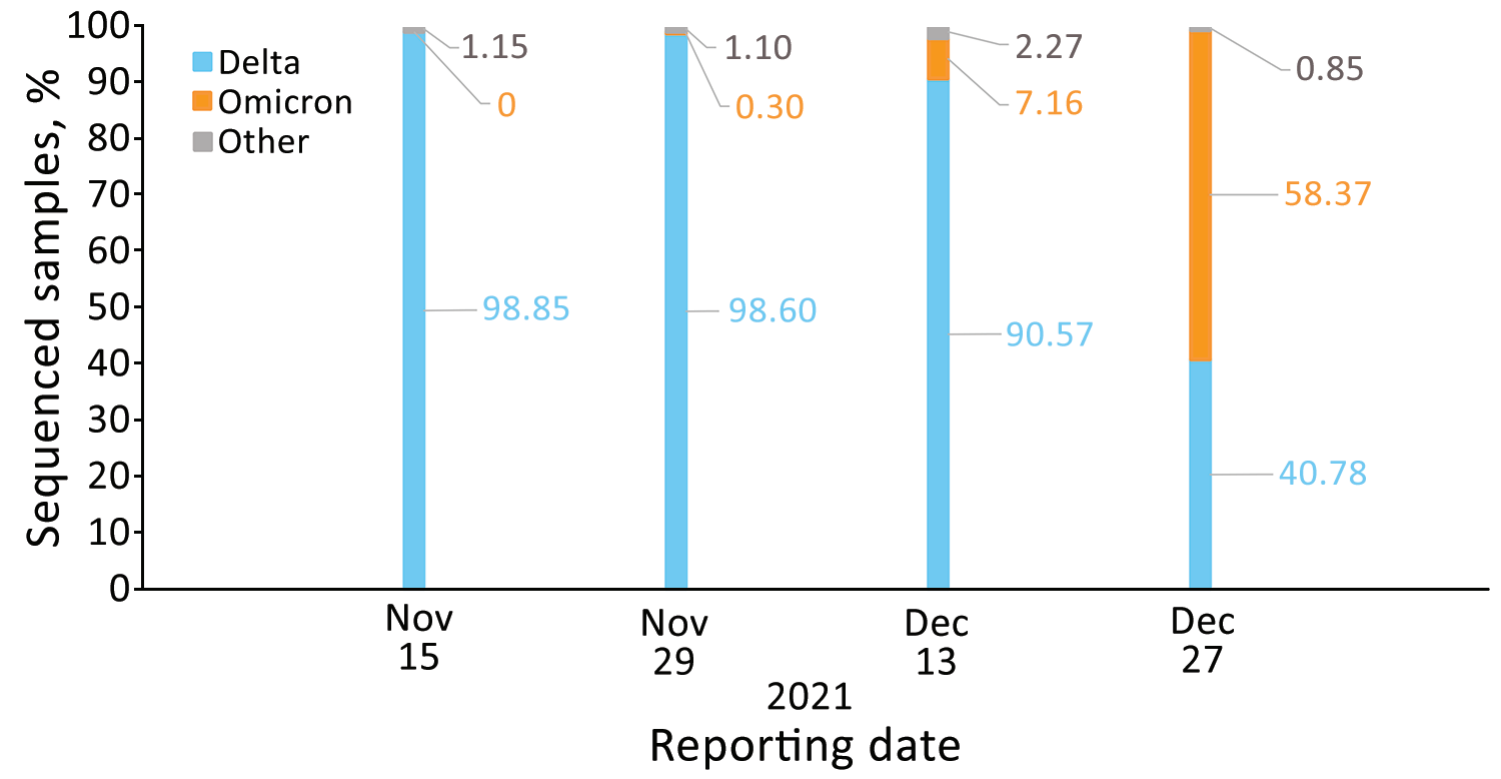
Percentage of sequenced severe acute respiratory syndrome coronavirus 2 samples by variant and reporting date, Israel, November 15, November 29, December 13, and December 27, 2021. Based on (*9*). Numbers within the figure represent percentages of sequenced samples.

We excluded residents of Israel who tested positive for SARS-CoV-2 by PCR before the evaluation periods from the analyses (Appendix Table 1, Figures 2, 3). We estimated VE for the 16–59-year and >60-year age groups, as well as for age groups 16–29 years, 30–39 years, 40–49 years, and 50–59 years (Appendix Figures 2, 3). We first estimated VE for each cohort starting week 2 after the index date. We then estimated VE for all 14 cohorts combined. Because of the different index dates for these age groups, we followed persons 16–59 years of age for 16 weeks and persons >60 years of age for 20 weeks (Figure 1, panels C, D; Appendix Table 2).

### Hospitalizations, Severe Disease, and Death among SARS-CoV-2–Positive Booster Dose Recipients

We determined rates of SARS-CoV-2–related hospitalizations, severe or critical disease, and deaths for booster-dose recipients and for unvaccinated persons who tested positive for SARS-CoV-2 by PCR during the evaluation period described previously (breakthrough cases). The time allotted for the occurrence of hospitalization and severe or critical disease after the first positive PCR test was 14 days (*6*). We did not set a time limit for death after the first positive PCR test. We determined disease severity in accordance with US National Institutes of Health guidelines (*7*).

### Statistics

We determined VE and 95% CI using the formula (1 – incidence rate ratio [IRR]) × 100. The IRR represents the ratio of PCR-confirmed SARS-CoV-2 infection rate in the group of booster-dose recipients to the corresponding rate in the unvaccinated control group. For persons who tested positive for SARS-CoV-2 by PCR several times during the evaluation period, we included only the first positive test result in the analysis.

We excluded persons who had a positive SARS-CoV-2 PCR test before the evaluation periods from the analysis, regardless of their vaccination status. Unvaccinated persons included in the study who were vaccinated during the cohort evaluation period were censored (removed from the study) on their vaccination dates.

We computed the number of unvaccinated controls by age and sex for each cohort by subtracting the number of residents of Israel, by age and sex, who were vaccinated with any number of BNT162b2 vaccine doses before or on the cohort vaccination date (index date) from the number of residents who did not have a recorded positive SARS-CoV-2 PCR test by that date. We calculated the number of person-days each person contributed as unvaccinated during each evaluation period. The number of residents (total, by age and by sex) was based on the 2021 Central Bureau of Statistics statistical abstract (*8*). We took into account unvaccinated participants who were included in >1 cohort when calculating VEs and CIs.

VE was first calculated for each age group daily cohort by week starting the second week after the booster dose. VE was estimated separately for each week that passed since the index date. For the combined VE estimate (for all 14 cohorts together), we took several steps. First, we summed the number of booster-vaccinated and unvaccinated SARS-CoV-2–positive cases for the evaluation period. Second, we counted the days at risk for each age-group cohort on the basis of the number of person-days for each booster-vaccinated and unvaccinated person from the start of the study until the person became SARS-CoV-2–positive or until the end of follow-up, whichever date was earlier. Third, we summed the days at risk for each age group cohort during the evaluation period to provide the total number of person-days at risk in the booster-vaccinated or unvaccinated status for all age group cohorts. Finally, we calculated IRR for the age group cohorts combined.

We evaluated the reduction in SARS-CoV-2–related hospitalizations, illness severity during hospitalizations, and death in persons who received 3 BNT162b2 vaccine doses compared with unvaccinated persons using the formula (1 – IRR) × 100. We performed adjustment of IRR and 95% CI for age group (16–29, 30–39, 40–49, and 50–59 years for persons 16–59 years of age; 60–79 and >80 years for persons >60 years of age), sex and epidemiologic week, provided the data sizes were sufficiently large, by using Poisson regression. Statistical analysis was performed using SAS Enterprise Guide 7.1 software (SAS Institute, https://www.sas.com). The study was approved by the superior ethical committee of the Israel MOH (protocol no. CoR-MOH-081–2021) with exemption from informed consent.

## Results

### Booster Dose Vaccination Campaign

By October 31, 2021, persons ≥60 years of age reached a vaccination rate of ≈80% (Appendix Figure 1). Vaccination rates by that date were 70.2% for the 50–59-year age group, 62.4% for the 40–49-year age group, 53.1% for the 30–39-year age group, and 44.7% for the 16–29-year age group (Appendix Figure 1).

### Booster Dose VE in Persons 16–59 Years of Age

Adjusted VE point estimates reached 92.8% (95% CI 91.3%–94.0%) in week 2 of the evaluation period and 96.8% (95% CI 96.0%–97.5%) by week 3 (Figure 2, panel A; Appendix Table 3). The adjusted VE remained above 95% until week 10 and thereafter started to slowly decline, reaching VE of 89.6% (95% CI 85.4%–92.7%) in week 14. In weeks 15 and 16, VE point estimates declined by 12%, reaching a point estimate of 77.6% (95% CI 68.4%–84.2%) (Figure 2, panel A; Appendix Table 3). The evaluation dates of weeks 15 and 16 occurred during December 2021 (Appendix Table 2), when the percentage of the B.1.1.529 (Omicron) variant among reported sequenced samples in Israel rapidly increased (Figure 3) (*9*). VE estimation by age groups demonstrated similar patterns (Appendix Figure 4).

### Booster Dose VE in Persons >60 Years of Age

Adjusted VE point estimates reached 76.4% (95% CI 70.9%–80.9%) on week 2 of the evaluation period and 93.1% (95% CI 91.8%–94.2%) by week 3 (Figure 2, panel B; Appendix Table 3). The adjusted VE remained above 93% until week 13, and thereafter started to slowly decline, reaching VE of 90.6% (95% CI 87.2%–93.1%) at week 17. In weeks 18 and 19, VE point estimates declined by 7% and in week 20, VE declined by 21.6%, reaching a point estimate of 61.3% (95% CI 52.5%–68.4%) (Figure 2, panel B; Appendix Table 3). The evaluation dates of weeks 19 and 20 occurred during December 2021 (Appendix Table 2), when the percentage of the B.1.1.529 (Omicron) variant among reported sequenced samples in Israel rapidly increased (Figure 3) (*9*).

### Hospitalizations among SARS-CoV-2–Positive Booster Dose Vaccine Recipients

We analyzed rate reductions of hospitalizations among persons who became SARS-CoV-2-positive by week and for all evaluation weeks combined (Table 1). The hospitalization rate reduction by week for persons 16–59 years of age was between 62.8% (95% CI −0.6% to 86.2%) and 100.0%. The combined rate reduction for weeks 2–16 was 89.2% (95% CI 79.1%–94.4%) (Table 1).

**Table 1:** Rate reduction of hospitalizations among SARS-CoV-2–positive persons who received the BNT162b2 COVID-19 vaccine booster dose, Israel*

Age group, y	Time of first positive SARS-CoV-2 PCR test after index date, wk	Unvaccinated SARS-CoV-2–positive persons			Vaccinated SARS-CoV-2–positive persons		Adjusted 1 – IRR, % (95% CI)†
		Hospitalized	Total		Hospitalized	Total	
16–59	2	889	22,545		4	1,343	92.2 (77.9–97.3)
	3	770	18,232		3	455	83.9 (46.7–95.1)
	4	590	12,962		2	293	84.6 (47.1–95.5)
	5	414	8,555		1	210	90.1 (44.5–98.2)
	6	259	5,531		1	138	84.3 (15.1–97.1)
	7	175	3,573		0	131	100.0
	8	120	2,626		1	83	74.0 (−52.5 to 95.6)
	9	92	2,013		0	54	100.0
	10	72	1,714		0	57	100.0
	11	69	1,401		0	66	100.0
	12	68	1,385		0	90	100.0
	13	72	1,442		0	118	100.0
	14	62	1,569		1	154	84.4 (5.9–97.4)
	15	64	2,145		4	351	62.8 (−0.6 to 86.2)
	16	71	4,088		1	884	94.0 (47.9–99.3)
	2–16 combined	1,662	41,135		18	4,427	89.2 (79.1–94.4)
>60	2	644	1,985		98	1,314	77.5 (71.5–82.3)
	3	666	2,200		39	464	73.0 (64.4–79.5)
	4	647	2,160		36	375	68.2 (52.7–78.6)
	5	619	2,164		25	319	73.2 (48.2–86.2)
	6	593	2,033		20	296	77.3 (62.5–86.3)
	7	501	1,724		29	271	64.5 (50.2–74.7)
	8	375	1,276		23	221	65.3 (40.2–79.9)
	9	254	869		6	147	86.7 (72.6–93.6)
	10	163	577		10	86	64.1 (27.1–82.3)
	11	108	376		9	63	54.9 (−35.9 to 85.1)
	12	80	258		4	44	71.0 (21.5–89.3)
	13	56	208		3	52	80.2 (54.4–91.4)
	14	56	183		3	49	81.9 (45.8–94.0)
	15	58	170		6	47	67.5 (32.6–84.4)
	16	53	162		5	44	65.7 (23.4–84.6)
	17	46	151		1	56	94.4 (57.3–99.2)
	18	42	147		1	69	95.0 (72.1–99.1)
	19	38	184		5	125	82.6 (57.7–92.8)
	20	46	305		13	432	81.4 (67.0–89.5)
	2–20 combined	1,976	6,673		336	4,474	75.1 (71.3–78.5)

*COVID-19, coronavirus disease; IRR, incidence rate ratio; SARS-CoV-2, severe acute respiratory syndrome coronavirus 2. †Adjusted for sex and epidemiologic week.

The hospitalization rate reduction by week for persons >60 years of age was between 54.9% (95% CI −35.9 to 85.1%) and 95.0% (95% CI 72.1%–99.1%). The combined rate reduction for weeks 2–20 was 75.1% (95% CI 71.3%–78.5%) (Table).

### Severe Disease among SARS-CoV-2–Positive Booster Dose Vaccine Recipients

The severe or critical disease rate reduction for persons 16–59 years of age was 92.0% (95% CI 70.0%–97.9%) on week 2 (Table 2). No cases of severe or critical disease were recorded among booster-dose recipients for weeks 3–16. The combined rate reduction for weeks 2–16 was 97.3% (95% CI 89.7%–99.3%) (Table 2).

**Table 2:** Rate reduction of severe or critical disease among SARS-CoV-2–positive persons who received the BNT162b2 COVID-19 vaccine booster dose, Israel*

Age group, y	Time of first positive SARS-CoV-2 PCR test after index date, wk	Unvaccinated SARS-CoV-2–positive persons			Vaccinated SARS-CoV-2–positive persons		Adjusted 1 – IRR, % (95% CI)†
		Severe or critical disease	Total		Severe or critical disease	Total	
16–59	2	422	22,545		2	1,343	92.0 (70.0–97.9)
	3	358	18,232		0	455	100.0
	4	262	12,962		0	293	100.0
	5	170	8,555		0	210	100.0
	6	113	5,531		0	138	100.0
	7	63	3,573		0	131	100.0
	8	40	2,626		0	83	100.0
	9	32	2,013		0	54	100.0
	10	27	1,714		0	57	100.0
	11	29	1,401		0	66	100.0
	12	24	1,385		0	90	100.0
	13	25	1,442		0	118	100.0
	14	21	1,569		0	154	100.0
	15	30	2,145		0	351	100.0
	16	30	4,088		0	884	100.0
	2–16 combined	727	41,135		2	4,427	97.3 (89.7–99.3)
>60	2	450	1,985		56	1,314	81.9 (75.4–86.7)
	3	470	2,200		27	464	73.5 (61.0–82.0)
	4	464	2,160		18	375	77.8 (63.1–86.8)
	5	469	2,164		15	319	78.9 (66.8–86.6)
	6	447	2,033		9	296	86.6 (74.2–93.0)
	7	390	1,724		12	271	81.4 (63.1–90.6)
	8	299	1,276		13	221	75.6 (54.1–87.0)
	9	207	869		2	147	94.6 (85.3–98.0)
	10	128	577		9	86	58.6 (11.1–80.7)
	11	78	376		2	63	86.2 (39.1–96.9)
	12	52	258		3	44	69.0 (−15.3 to 91.7)
	13	40	208		1	52	91.0 (63.8–97.8)
	14	42	183		2	49	84.0 (44.8–95.3)
	15	45	170		3	47	78.5 (49.3–90.8)
	16	37	162		3	44	70.3 (30.0–87.4)
	17	32	151		1	56	91.7 (43.4–98.8)
	18	28	147		0	69	100.0
	19	33	184		4	125	83.6 (47.0–94.9)
	20	42	305		7	432	89.0 (75.8–95.0)
	2–20 combined	1,465	6,673		187	4,474	81.6 (78.3–84.3)

*COVID-19, coronavirus disease; IRR, incidence rate ratio; SARS-CoV-2, severe acute respiratory syndrome coronavirus 2. †Adjusted for sex and epidemiologic week.

The rate reduction of severe or critical disease by week for persons >60 years of age was between 58.6% (95% CI 11.1%–80.7%) and 100%. The combined rate reduction for weeks 2–20 was 81.6% (95% CI 78.3%–84.3%) (Table 2).

### Deaths among SARS-CoV-2-Positive Booster Dose Vaccine Recipients

No deaths were recorded among booster-dose recipients 16–59 years of age during the evaluation weeks, compared with 1–45 deaths per week in the unvaccinated group, a rate reduction of 100% (Table 3). The death rate reduction by week for persons >60 years of age was between 49.1% (95% CI −44.3% to 82.1%) and 100%. The combined rate reduction for weeks 2–20 was 77.1% (95% CI 71.2%–81.8%) (Table 3). Analysis of death rate reduction by using only deaths that were highlighted by hospitals as deaths caused by COVID-19 and limiting the time from positive PCR test to death by up to 28 days yielded similar results (Appendix Tables 4, 5).

**Table 3:** Rate reduction of deaths among SARS-CoV-2–positive persons who received the BNT162b2 COVID-19 vaccine booster dose, Israel*

Age group, y	Time of first positive SARS-CoV-2 PCR test after index date, wk	Unvaccinated SARS-CoV-2–positive persons			Vaccinated SARS-CoV-2–positive persons		Adjusted 1 – IRR, % (95% CI)†
		Deaths	Total		Deaths	Total	
16–59	2	45	22,545		0	1,343	100
	3	36	18,232		0	455	100
	4	27	12,962		0	293	100
	5	17	8,555		0	210	100
	6	11	5,531		0	138	100
	7	6	3,573		0	131	100
	8	5	2,626		0	83	100
	9	4	2,013		0	54	100
	10	3	1,714		0	57	100
	11	3	1,401		0	66	100
	12	3	1,385		0	90	100
	13	2	1,442		0	118	100
	14	1	1,569		0	154	100
	15	1	2,145		0	351	100
	16	1	4,088		0	884	100
	2–16 combined	72	41,135		0	4,427	100
>60	2	243	1,985		31	1,314	81.9 (70.4–88.9)
	3	246	2,200		13	464	76.0 (55.3–87.2)
	4	226	2,160		13	375	68.1 (49.6–79.8)
	5	229	2,164		11	319	68.7 (42.7–82.9)
	6	201	2,033		7	296	76.5 (55.9–87.4)
	7	166	1,724		8	271	70.5 (29.4–87.7)
	8	122	1,276		8	221	63.1 (29.4–80.7)
	9	89	869		2	147	87.6 (53.7–96.7)
	10	59	577		5	86	49.1 (−44.3 to 82.1)
	11	30	376		0	63	100.0
	12	19	258		1	44	67.7 (−189.8 to 96.4)
	13	19	208		1	52	78.7 (−89.9 to 97.6)
	14	22	183		1	49	85.0 (8.5–97.5)
	15	21	170		1	47	85.3 (−21.3 to 98.2)
	16	17	162		0	44	100.0
	17	11	151		1	56	74.6 (−225.9 to 98.0)
	18	10	147		0	69	100.0
	19	7	184		2	125	56.5 (−144.3 to 92.3)
	20	8	305		3	432	70.7 (−2.7 to 91.7)
	2–20 combined	686	6,673		108	4,474	77.1 (71.2–81.8)

*COVID-19, coronavirus disease; IRR, incidence rate ratio; SARS-CoV-2, severe acute respiratory syndrome coronavirus 2. †Adjusted for sex and epidemiologic week.

## Discussion

Our results demonstrate that, after the BNT16b2 booster dose, VE against SARS-CoV-2 infection reached levels that were observed shortly after the second vaccine dose (*6*). VE point estimates of >90% were observed in week 2 in persons 16–59 years of age and in week 3 in persons >60 years of age. Similar delay in achieving high VE among elderly persons was also shown after the second BNT162b2 vaccine dose (*6*). Highest-level VE was maintained for up to 11 weeks, as shown in persons >60 years of age included in our study. The decline in VE that occurred afterward was initially mild, still maintaining VE point estimates >90% for up to week 17 of the evaluation period in persons >60 years of age. The decline in VE became steeper during the last 2 weeks of the evaluation period.

 The B.1.617.2 (Delta) variant was the most prevalent variant in Israel through November 2021. However, the last 2 evaluation weeks, which occurred in December 2021 (Appendix Table 2), coincided with the beginning of a new wave of illness and the sharp rise in the B.1.1.529 (Omicron) variant in Israel. Waning immunity was shown several months after the second BNT162b2 vaccine dose (*2*,*3*,*10*) and was temporarily associated with the rise of the B.1.617.2 (Delta) variant in Israel. However, a fresh 2-dose BNT162b2 vaccination regimen was found to be highly effective against the B.1.617.2 (Delta) variant (*1*).

Early evaluations suggest that VE of 2 doses of the BNT162b2 against B.1.1.529 (Omicron) variant–related infection, symptomatic disease, and hospitalizations was reduced compared with VE against the B.1.617.2 (Delta) variant (*11**,**12*; C.H. Hansen et al., unpub. data, https://www.medrxiv.org/content/10.1101/2021.12.20.21267966v3; N. Andrews et al., unpub. data, https://www.medrxiv.org/content/10.1101/2021.12.14.21267615v1). VE of the BNT162b2 booster dose against infection and symptomatic disease caused by the B.1.1.529 (Omicron) variant was also lower than for the B.1.617.2 (Delta) variant (*11*; C.H. Hansen et al., unpub. data; N. Andrews et al., unpub. data). The difference in VE against the B.1.1.529 (Omicron) and B.1.617.2 (Delta) variants increased as time passed from booster dose administration (*11*). Therefore, the steeper decrease in the 3-dose VE in the last 2 weeks of our study period could be caused, at least in part, by the rapid spread of the B.1.1.529 (Omicron) variant in Israel.

Several studies have evaluated the shorter-term effect of the BNT162b2 booster dose on SARS-CoV-2 infection and complications (*13**–**17*; N. Andrews et al., unpub. data, https://www.medrxiv.org/content/10.1101/2021.11.15.21266341v1) and found that a high degree of protection was achieved. Some of these studies used booster-eligible 2-dose vaccine recipients as controls (*13*–*15*,*17*), but our study evaluated VE by using unvaccinated persons as controls. Booster dose VE analysis using unvaccinated persons as controls is paramount, because the baseline VE against SARS-CoV-2 for booster dose recipient is >0%, and time to eligibility for a booster dose might vary among countries. Furthermore, our analysis shows the magnitude of protection offered by the booster dose in a manner that enables easy comparison with other VE studies.

Analyzing the reduction in complications among SARS-CoV-2 vaccine recipients is crucial for public health policy. Our results demonstrated substantial protection from complications among booster-dose vaccine recipients throughout the evaluation period and, further, suggest that this protection may be higher than the protection found shortly after the receipt of the second dose (*6*). Although a study from a health maintenance organization in Israel demonstrated VE estimates of 93% against hospitalizations, 92% against severe disease, and 81% against death (*15*), such analysis cannot distinguish between complications averted because of reductions in SARS-CoV-2 infections and reduction of complications among breakthrough cases. Further analysis is necessary to determine whether rate reductions of complications in booster-dose recipients are affected by the spread of the B.1.1.529 (Omicron) variant and whether those rate reductions are waning over time.

Our study’s first limitation is that the size of the unvaccinated control study group was calculated on the basis of Israel Central Bureau of Statistics data. Nevertheless, these data included population size by sex and age, which enables statistical adjustment. Furthermore, data concerning hospitalizations, disease severity, and deaths were available in the SARS-CoV-2 PCR test repository for unvaccinated SARS-CoV-2–positive persons. The lack of information regarding the presence of comorbidities constitutes another limitation. However, the use of multiple cohorts, the size of the population included in our study, the consistent VE estimates among various age groups, and the successful use of similar methodology in previous SARS-CoV-2 VE studies (*1*,*6*) support the validity of our results.

In this evaluation, we did not estimate VE against symptomatic disease. When the number of PCR-positive persons increases, the ability to conduct epidemiologic investigation and determine whether symptoms were present greatly diminishes. A further limitation was the low number of weekly complications in SARS-CoV-2–positive persons, particularly in weeks of lower SARS-CoV-2 circulation (Tables 1–3). However, this limitation was less evident among persons >60 years of age, for whom the number of weekly complications is higher than for persons 16–59 years of age.

SARS-CoV-2 PCR testing and vaccination practices could vary among persons. Such differences can stem from behavior, occupation (such as being a healthcare worker), or health factors (such as having symptoms or risk factors or residing in a nursing home) and can potentially affect VE estimates against infection. Because SARS-CoV-2 PCR testing has been commonly performed among hospitalized patients, determination of reductions in hospitalizations, severe or critical disease, and death rate were probably not affected by factors that might affect testing practices of nonhospitalized patients.

No distinction was available in the MOH SARS-CoV-2 data repository between persons who were hospitalized because of COVID-19 and those who were hospitalized because of other reasons and were SARS-CoV-2–positive. However, the severity status that is registered in the repository is given to COVID-19 patients on the basis of National Institutes of Health guidelines (*7*).

In conclusion, our results showing high VE of the BNT162b2 booster dose against SARS-CoV-2 cases and the maintenance of positive effects among breakthrough cases demonstrate the duration of the booster-dose effect during a period in which the Delta variant was predominant. However, the reduced VE in an Omicron-variant setting indicates that additional tools are required to combat new variants of concern.

Appendix Additional information about effectiveness of BNT162b2 vaccine booster against SARS-CoV-2 infection and breakthrough complications, Israel.

## References

[1] Glatman-Freedman A, Hershkovitz Y, Kaufman Z, Dichtiar R, Keinan-Boker L, Bromberg M. Effectiveness of BNT162b2 vaccine in adolescents during outbreak of SARS-CoV-2 Delta variant infection, Israel, 2021. Emerg Infect Dis. 2021;27:2919–22. 10.3201/eid2711.211886 34570694 PMC8544958

[2] Goldberg Y, Mandel M, Bar-On YM, Bodenheimer O, Freedman L, Haas EJ, et al. Waning immunity after the BNT162b2 vaccine in Israel. N Engl J Med. 2021;385:e85. 10.1056/NEJMoa2114228 34706170 PMC8609604

[3] Levin EG, Lustig Y, Cohen C, Fluss R, Indenbaum V, Amit S, et al. Waning immune humoral response to BNT162b2 COVID-19 vaccine over 6 months. N Engl J Med. 2021;385:e84. 10.1056/NEJMoa2114583 34614326 PMC8522797

[4] Israel Ministry of Health. The vaccination advisory committee presented data and recommended the administration of a third dose to older adults. 2021 Jul 30 [cited 2021 Nov 21]. https://www.gov.il/en/departments/news/29072021-04

[5] Israel Ministry of Health. Discussions on administering the third vaccine to additional populations. 2021 Aug 20 [cited 2021 Nov 21]. https://www.gov.il/en/departments/news/19082021-04

[6] Glatman-Freedman A, Bromberg M, Dichtiar R, Hershkovitz Y, Keinan-Boker L. The BNT162b2 vaccine effectiveness against new COVID-19 cases and complications of breakthrough cases: A nation-wide retrospective longitudinal multiple cohort analysis using individualised data. EBioMedicine. 2021;72:103574. 10.1016/j.ebiom.2021.103574 34537449 PMC8445746

[7] National Institutes of Health. Coronavirus disease 2019 (COVID-19) treatment guidelines: clinical spectrum of SARS-CoV-2 infection [cited 2021 Feb 25]. https://www.covid19treatmentguidelines.nih.gov 34003615

[8] Central Bureau of Statistics. Statistical abstract of Israel 2021—no. 72 [cited 2021 Nov 22]. https://www.cbs.gov.il/en/publications/Pages/2021/Population-Statistical-Abstract-of-Israel-2021-No.72.aspx

[9] Our World in Data. SARS-CoV-2 variants in in analyzed sequences, Israel. 2022 [cited 2022 March 6]. https://ourworldindata.org/grapher/covid-variants-area?country=~ISR

[10] Bayart JL, Douxfils J, Gillot C, David C, Mullier F, Elsen M, et al. Waning of IgG, total and neutralizing antibodies 6 months post-vaccination with BNT162b2 in healthcare workers. Vaccines (Basel). 2021;9:1092. 10.3390/vaccines9101092 34696200 PMC8540417

[11] UK Health Security Agency. SARS-CoV-2 variants of concern and variants under investigation in England. Technical briefing 33. 2021 [cited 2022 Jan 9]. https://assets.publishing.service.gov.uk/government/uploads/system/uploads/attachment_data/file/1043807/technical-briefing-33.pdf

[12] Collie S, Champion J, Moultrie H, Bekker LG, Gray G. Effectiveness of BNT162b2 vaccine against Omicron variant in South Africa. N Engl J Med. 2022;386:494–6. 10.1056/NEJMc2119270 34965358 PMC8757569

[13] Bar-On YM, Goldberg Y, Mandel M, Bodenheimer O, Freedman L, Kalkstein N, et al. Protection of BNT162b2 vaccine booster against COVID-19 in Israel. N Engl J Med. 2021;385:1393–400. 10.1056/NEJMoa2114255 34525275 PMC8461568

[14] Bar-On YM, Goldberg Y, Mandel M, Bodenheimer O, Freedman L, Alroy-Preis S, et al. Protection against COVID-19 by BNT162b2 booster across age groups. N Engl J Med. 2021;385:2421–30. 10.1056/NEJMoa2115926 34879188 PMC8728796

[15] Barda N, Dagan N, Cohen C, Hernán MA, Lipsitch M, Kohane IS, et al. Effectiveness of a third dose of the BNT162b2 mRNA COVID-19 vaccine for preventing severe outcomes in Israel: an observational study. Lancet. 2021;398:2093–100. 10.1016/S0140-6736(21)02249-2 34756184 PMC8555967

[16] Tartof SY, Slezak JM, Puzniak L, Hong V, Frankland TB, Ackerson BK, et al. Effectiveness of a third dose of BNT162b2 mRNA COVID-19 vaccine in a large US health system: a retrospective cohort study. Lancet Reg Health Am. 2022 Feb 14 [Epub ahead of print]. 10.1016/j.lana.2022.100198 PMC8841530 35187521

[17] Andrews N, Stowe J, Kirsebom F, Toffa S, Sachdeva R, Gower C, et al. Effectiveness of COVID-19 booster vaccines against covid-19 related symptoms, hospitalisation and death in England. Nat Med. 2022; [Epub ahead of print]. 10.1038/s41591-022-01699-1 PMC9018410 35045566

