# Supplementary material for: Effectiveness of BNT162b2 Vaccine Booster against SARS-CoV-2 Infection and Breakthrough Complications, Israel
Source: Emerg Infect Dis. 2022 May;28(5):948–56. doi: 10.3201/eid2805.220141 (PMC9045419; doi:10.3201/eid2805.220141)
Supplement: Appendix — Additional information about effectiveness of BNT162b2 vaccine booster against SARS-CoV-2 infection and breakthrough complications, Israel. [file 22-0141-Techapp-s1.pdf]

Article DOI: https://doi.org/10.3201/eid2805.220141

Effectiveness of BNT162b2 Vaccine Booster against SARS-CoV-2 Infection and Breakthrough Complications, Israel

Appendix

Appendix Table 1. Inclusion and exclusion criteria of BNT162b2 booster dose recipients

Criteria	
Inclusion criteria for vaccinated persons included in vaccine effectiveness analysis	
1	Persons who received the BNT162b2 booster dose on the index dates
Exclusion criteria for vaccinated persons included in vaccine effectiveness analysis	
1	Persons who were SARS-CoV-2–positive before the evaluation periods

Appendix Table 2. Dates of evaluation weeks by age group in study of effectiveness of BNT162b2 vaccine booster against SARS-CoV-2 infection, Israel*

	16–59 y (Index dates August 29–September 11, 2021)		≥60 y (Index dates August 1–September 14, 2021)	
Evaluation week after booster dose	Evaluation week start date	Evaluation week end date	Evaluation week start date	Evaluation week end Date
2	Sep 6, 2021	Sep 25, 2021	Aug 9, 2021	Aug 28, 2021
3	Sep 13, 2021	Oct 2, 2021	Aug 16, 2021	Sep 4, 2021
4	Sep 20, 2021	Oct 9, 2021	Aug 23, 2021	Sep 11, 2021
5	Sep 27, 2021	Oct 16, 2021	Aug 30, 2021	Sep 18, 2021
6	Oct 4, 2021	Oct 23, 2021	Sep 6, 2021	Sep 25, 2021
7	Oct 11, 2021	Oct 30, 2021	Sep 13, 2021	Oct 2, 2021
8	Oct 18, 2021	Nov 6, 2021	Sep 20, 2021	Oct 9, 2021
9	Oct 25, 2021	Nov 13, 2021	Sep 27, 2021	Oct 16, 2021
10	Nov 1, 2021	Nov 20, 2021	Oct 4, 2021	Oct 23, 2021
11	Nov 8, 2021	Nov 27, 2021	Oct 11, 2021	Oct 30, 2021
12	Nov 15, 2021	Dec 4, 2021	Oct 18, 2021	Nov 6, 2021
13	Nov 22, 2021	Dec 11, 2021	Oct 25, 2021	Nov 13, 2021
14	Nov 29, 2021	Dec 18, 2021	Nov 1, 2021	Nov 20, 2021
15	Dec 6, 2021	Dec 25, 2021	Nov 8, 2021	Nov 27, 2021
16	Dec 13, 2021	Jan 1, 2022	Nov 15, 2021	Dec 4, 2021
17	NA	NA	Nov 22, 2021	Dec 11, 2021
18	NA	NA	Nov 29, 2021	Dec 18, 2021
19	NA	NA	Dec 6, 2021	Dec 25, 2021
20	NA	NA	Dec 13, 2021	Jan 1, 2022

*NA, not applicable; SARS-CoV-2, severe acute respiratory syndrome coronavirus 2.

Appendix Table 3. Effectiveness of BNT162B2 vaccine booster against SARS-CoV-2 infection by week since index date*

		Unvaccinated		Vaccinated		
Age group, y	Time of first positive SARS-CoV-2 PCR test after index date, wk	SARS-CoV-2–positive cases	Person-days	SARS-CoV-2–positive cases	Person-days	Adjusted VE (95% CI)†
16–59	2	22,545	8,578,224	1,343	5,533,381	92.8% (91.3 to 94.0)
16–59	3	18,232	8,352,499	455	5,527,946	96.8% (96.0 to 97.5)
16–59	4	12,962	8,147,548	293	5,525,303	97.4% (96.5 to 98.0)
16–59	5	8,555	7,960,333	210	5,523,483	97.3% (96.5 to 97.9)
16–59	6	5,531	7,803,622	138	5,522,291	97.0% (95.9 to 97.8)
16–59	7	3,573	7,692,617	131	5,521,323	95.7% (94.1 to 96.8)
16–59	8	2,626	7,605,652	83	5,520,557	95.9% (94.2 to 97.1)

Age group, y	Time of first positive SARS-CoV-2 PCR test after index date, wk	Unvaccinated SARS-CoV-2–positive cases	Unvaccinated Person-days	Vaccinated SARS-CoV-2–positive cases	Vaccinated Person-days	Adjusted VE (95% CI)†
16–59	9	2,013	7,528,652	54	5,520,093	96.1% (94.1 to 97.4)
16–59	10	1,714	7,461,302	57	5,519,689	95.5% (93.2 to 97.0)
16–59	11	1,401	7,404,380	66	5,519,268	93.2% (89.9 to 95.4)
16–59	12	1,385	7,352,260	90	5,518,739	92.5% (89.1 to 94.8)
16–59	13	1,442	7,302,363	118	5,518,067	90.9% (87.1 to 93.6)
16–59	14	1,569	7,225,588	154	5,517,134	89.6% (85.4 to 92.7)
16–59	15	2,145	7,171,183	351	5,515,601	84.0% (77.3 to 88.7)
16–59	16	4,088	7,103,771	884	5,511,898	77.6% (68.4 to 84.2)
≥60	2	1,985	1,718,310	1,314	5,529,468	76.4% (70.9 to 80.9)
≥60	3	2,200	1,690,156	464	5,523,847	93.1% (91.8 to 94.2)
≥60	4	2,160	1,663,298	375	5,520,982	94.9% (94.2 to 95.6)
≥60	5	2,164	1,637,562	319	5,518,575	95.7% (95.1 to 96.3)
≥60	6	2,033	1,614,522	296	5,516,364	95.1% (93.4 to 96.4)
≥60	7	1,724	1,593,029	271	5,514,354	95.6% (94.9 to 96.2)
≥60	8	1,276	1,573,462	221	5,512,578	93.9% (91.9 to 95.4)
≥60	9	869	1,555,278	147	5,511,296	95.4% (94.5 to 96.1)
≥60	10	577	1,518,819	86	5,510,446	96.0% (95.0 to 96.8)
≥60	11	376	1,500,575	63	5,509,937	95.5% (94.1 to 96.6)
≥60	12	258	1,470,086	44	5,509,550	95.1% (93.2 to 96.5)
≥60	13	208	1,449,489	52	5,509,246	93.6% (91.3 to 95.3)
≥60	14	183	1,427,719	49	5,508,902	92.3% (89.4 to 94.4)
≥60	15	170	1,423,875	47	5,508,558	91.7% (82.4 to 96.1)
≥60	16	162	1,444,306	44	5,508,227	93.0% (90.2 to 95.0)
≥60	17	151	1,418,856	56	5,507,900	90.6% (87.2 to 93.1)
≥60	18	147	1,404,861	69	5,507,427	87.5% (83.2 to 90.6)
≥60	19	184	1,404,773	125	5,506,868	82.9% (77.4 to 87.0)
≥60	20	305	1,443,791	432	5,505,404	61.3% (52.5 to 68.4)

*SARS-CoV-2, severe acute respiratory syndrome coronavirus 2.
†Adjusted for age, sex, and epidemiologic week.

Appendix Table 4. Rate reduction of deaths caused by COVID-19 among persons who received the BNT162b2 vaccine booster dose, Israel*

Age group, y	Time of first positive SARS-CoV-2 PCR test after index date, wks	Unvaccinated SARS-CoV-2–positive persons: Deaths	Unvaccinated SARS-CoV-2–positive persons: Total	Vaccinated SARS-CoV-2–positive persons: Deaths	Vaccinated SARS-CoV-2–positive persons: Total	Adjusted 1-IRR (95% CI)†
16–59	2	41	22,545	0	1,343	100%
16–59	3	35	18,232	0	455	100%
16–59	4	23	12,962	0	293	100%
16–59	5	13	8,555	0	210	100%
16–59	6	7	5,531	0	138	100%
16–59	7	6	3,573	0	131	100%
16–59	8	5	2,626	0	83	100%
16–59	9	4	2,013	0	54	100%
16–59	10	1	1,714	0	57	100%
16–59	11	1	1,401	0	66	100%
16–59	12	1	1,385	0	90	100%
16–59	13	2	1,442	0	118	100%
16–59	14	1	1,569	0	154	100%
16–59	15	0	2,145	0	351	100%
16–59	16	0	4,088	0	884	100%
16–59	2–16 combined	61	41,135	0	4,427	100%
≥60	2	236	1,985	32	1,314	80.5% (68.3–88.0)
≥60	3	239	2,200	12	464	77.2% (56.3–88.1)
≥60	4	222	2,160	12	375	70.2% (51.0–81.8)
≥60	5	223	2,164	8	319	76.6% (57.7–87.1)
≥60	6	198	2,033	7	296	76.2% (56.5–87.0)
≥60	7	162	1,724	8	271	69.9% (27.6–87.5)
≥60	8	120	1,276	7	221	67.3% (29.9–84.8)
≥60	9	88	869	2	147	87.5% (52.0–96.8)
≥60	10	58	577	5	86	47.4% (−43.5–80.8)
≥60	11	29	376	0	63	100%
≥60	12	19	258	1	44	67.7% (−189.8–96.4)

Age group, y	Time of first positive SARS-CoV-2 PCR test after index date, wks	Unvaccinated SARS-CoV-2–positive persons		Vaccinated SARS-CoV-2–positive persons		Adjusted 1-IRR (95% CI)†
		Deaths	Total	Deaths	Total	
>60	13	19	208	1	52	78.7% (−89.9–97.6)
>60	14	22	183	1	49	85.0% (8.5–97.5)
>60	15	21	170	1	47	85.3% (−21.3–98.2)
>60	16	16	162	0	44	100%
>60	17	10	151	1	56	72.7% (−205.2–97.6)
>60	18	9	147	0	69	100%
>60	19	3	184	2	125	−31.1% (−864.9–82.2)
>60	20	3	305	0	432	100%
>60	2–20 combined	664	6,673	100	4,474	78.1% (71.8–82.9)

*COVID-19, coronavirus disease; SARS-CoV-2, severe acute respiratory syndrome coronavirus 2.
†Adjusted for sex and epidemiologic week.

Appendix Table 5. Rate reduction of deaths caused by COVID-19 among persons who received the BNT162b2 vaccine booster dose (for deaths that occurred within 28 d of positive test), Israel

Age group, y	Time of first positive SARS-CoV-2 PCR test after index date, wks	Unvaccinated SARS-CoV-2–positive persons		Vaccinated SARS-CoV-2–positive persons		Adjusted 1-IRR (95% CI)†
		Deaths	Total	Deaths	Total	
16–59	2	25	22,545	0	1,343	100%
16–59	3	22	18,232	0	455	100%
16–59	4	13	12,962	0	293	100%
16–59	5	9	8,555	0	210	100%
16–59	6	6	5,531	0	138	100%
16–59	7	3	3,573	0	131	100%
16–59	8	2	2,626	0	83	100%
16–59	9	2	2,013	0	54	100%
16–59	10	1	1,714	0	57	100%
16–59	11	1	1,401	0	66	100%
16–59	12	1	1,385	0	90	100%
16–59	13	2	1,442	0	118	100%
16–59	14	1	1,569	0	154	100%
16–59	15	0	2,145	0	351	100%
16–59	16	0	4,088	0	884	100%
16–59	2–16 combined	38	41,135	0	4,427	100%
>60	2	216	1,985	29	1,314	80.5% (67.1–88.4)
>60	3	211	2,200	12	464	73.8% (48.1–86.8)
>60	4	196	2,160	10	375	71.4% (44.6–85.2)
>60	5	196	2,164	8	319	73.1% (52.0–84.9)
>60	6	179	2,033	7	296	73.4% (51.8–85.3)
>60	7	149	1,724	7	271	71.1% (30.2–88.0)
>60	8	109	1,276	6	221	69.1% (29.8–86.4)
>60	9	75	869	2	147	85.3% (42.1–96.3)
>60	10	46	577	5	86	34.4% (−97.0–78.2)
>60	11	22	376	0	63	100%
>60	12	17	258	1	44	63.4% (−188.1–95.3)
>60	13	16	208	1	52	70.6% (−57.5–94.5)
>60	14	19	183	1	49	80.9% (3.5–96.2)
>60	15	17	170	1	47	79.9% (−49.8–97.3)
>60	16	15	162	0	44	100%
>60	17	10	151	1	56	72.7% (−205.2–97.6)
>60	18	9	147	0	69	100%
>60	19	3	184	2	125	−31.1% (−864.9–82.2)
>60	20	3	305	0	432	100%
>60	2–20 combined	593	6,673	93	4,474	77.0% (70.3–82.2)

*COVID-19, coronavirus disease; SARS-CoV-2, severe acute respiratory syndrome coronavirus 2.
†Adjusted for sex and epidemiologic week.

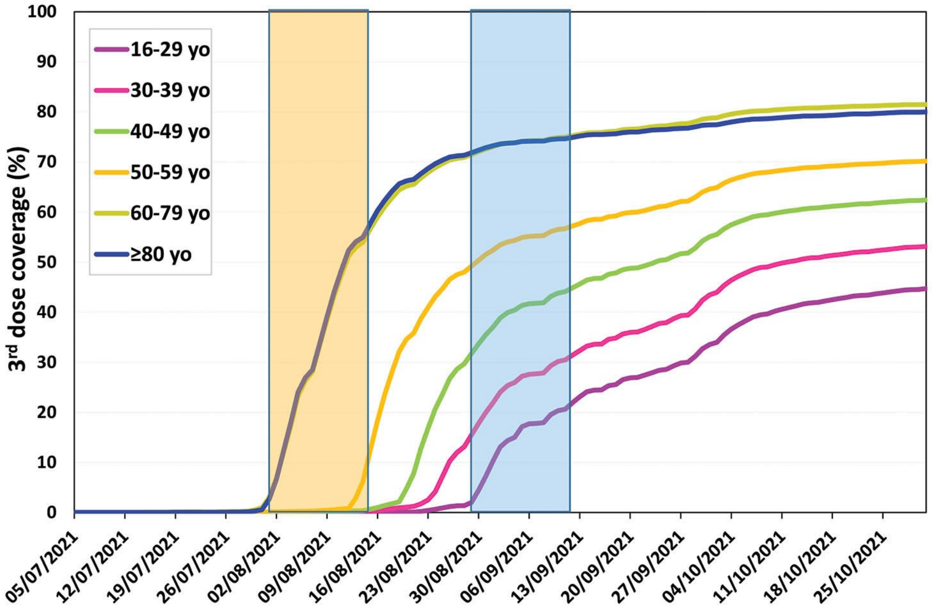

Appendix Figure 1. Coverage of third (booster) dose of COVID-19 BNT162b2 vaccine (Pfizer-BioNTech, https://www.pfizer.com), by age group, Israel, 2021. Orange area highlights the index dates for the cohorts that evaluate persons >60 years of age. Light blue area highlights the index dates for the cohorts that evaluate persons 16–59 years of age.

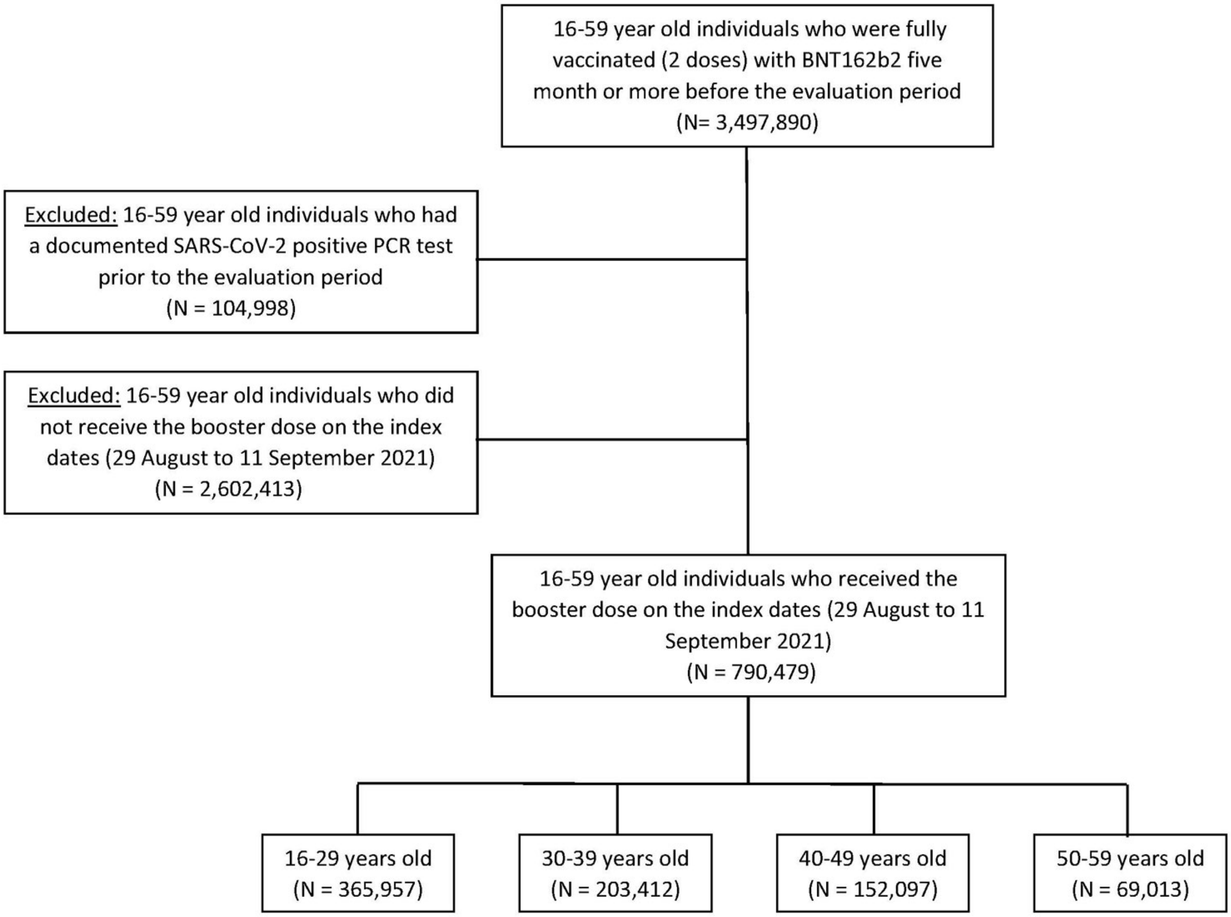

Appendix Figure 2. Flowchart of COVID-19 BNT162b2 vaccine (Pfizer-BioNTech, https://www.pfizer.com) booster dose recipients 16–59 years of age included in analysis of vaccine effectiveness (index dates August 29–September 11, 2021), Israel

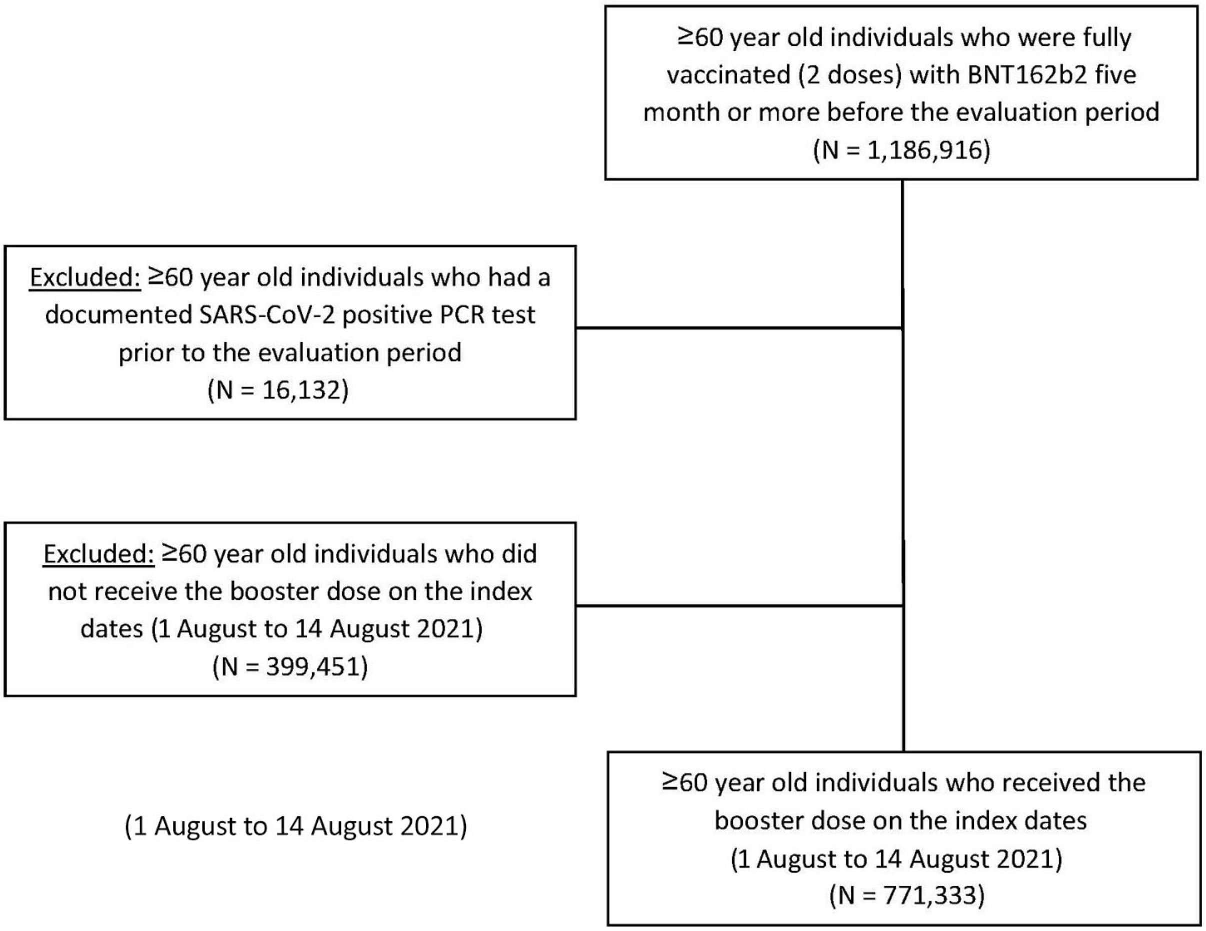

Appendix Figure 3. Flowchart of COVID-19 BNT162b2 vaccine (Pfizer-BioNTech, https://www.pfizer.com) booster dose recipients $\geq$60 years of age included in VE analysis (index dates August 1–August 14, 2021), Israel

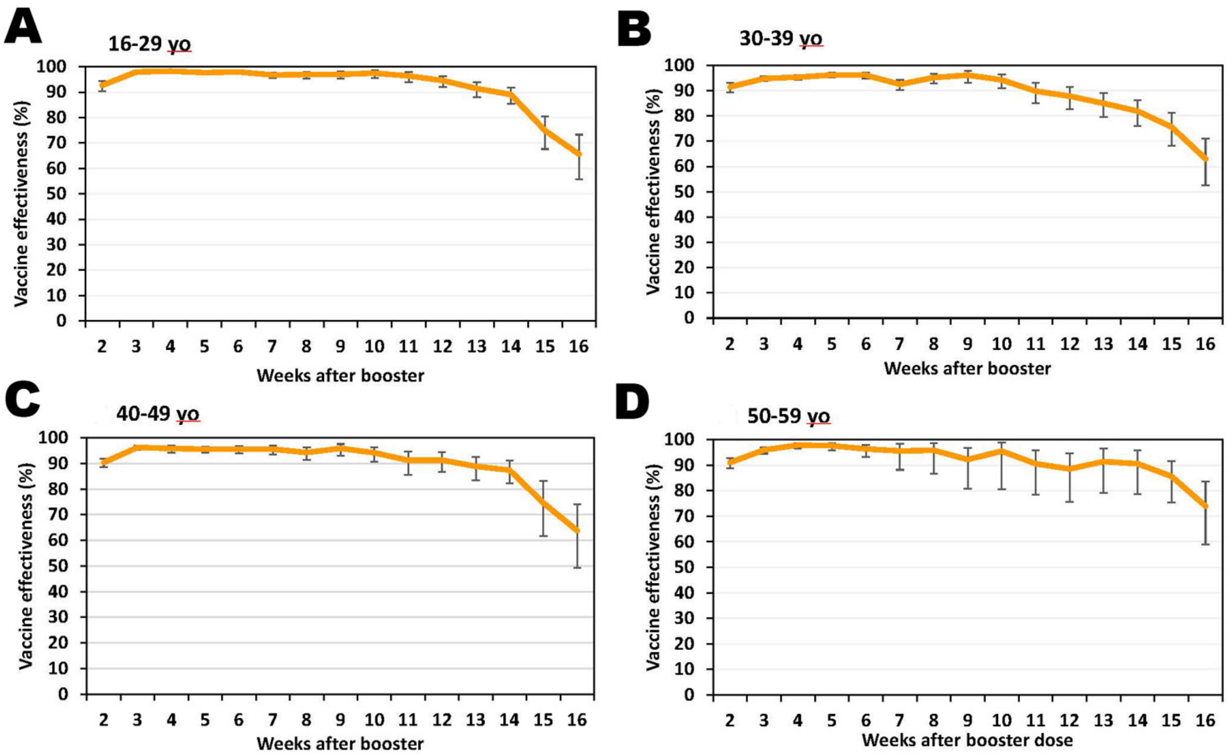

Appendix Figure 4. Adjusted vaccine effectiveness of COVID-19 BNT162b2 vaccine (Pfizer-BioNTech, https://www.pfizer.com) booster dose against SARS-CoV-2 infection by age group, adjusted for sex and epidemiologic week, Israel
